# Amplification of weak magnetic field effects on oscillating reactions

**DOI:** 10.1038/s41598-021-88871-8

**Published:** 2021-05-05

**Authors:** Thomas C. Player, Edward D. A. Baxter, Sarah Allatt, P. J. Hore

**Affiliations:** Department of Chemistry, University of Oxford, Physical and Theoretical Chemistry Laboratory, Oxford, OX1 3QZ UK

**Keywords:** Physical chemistry, Theoretical chemistry

## Abstract

We explore the possibility that chemical feedback and autocatalysis in oscillating chemical reactions could amplify weak magnetic field effects on the rate constant of one of the constituent reactions, assumed to proceed via a radical pair mechanism. Using the Brusselator model oscillator, we find that the amplitude of limit cycle oscillations in the concentrations of reaction intermediates can be extraordinarily sensitive to minute changes in the rate constant of the initiation step. The relevance of such amplification to biological effects of 50/60 Hz electromagnetic fields is discussed.

## Introduction

It is well established that magnetic fields (≥ 1 mT) can affect the kinetics and yields of certain chemical reactions even though the relevant interaction energies are orders of magnitude smaller than the thermal energy, *k*_B_*T*^1–5^. According to the radical pair mechanism, such effects arise from the electron spin-conserving nature of radical recombination reactions and the relatively long lifetimes of spin coherence in pairs of spin-correlated radicals. Both observed and predicted changes in reaction rates and/or product yields are normally a few tens of percent at most and often a good deal less, especially for magnetic fields weaker than 1 mT, leading to speculations about possible chemical amplification mechanisms^6–10^. Much of this debate has focussed on the weak geomagnetic fields (~ 50 μT) involved in avian magnetoreception^11–14^ and the even weaker anthropogenic 50/60 Hz electromagnetic fields (< 1 μT) that may or may not have adverse implications for human health^15–19^. It would therefore be interesting to know whether a small magnetically-induced change in the rate constant of one step in a multi-step chemical reaction could result in a disproportionately large change in either the concentrations of downstream species or the overall rate of the reaction.

In 1996, Eichwald and Wallaczek^20^ appeared to answer this question with the claim that chemical amplification could arise in the Michaelis–Menton (MM) model of enzyme kinetics if one of the three reaction steps were to proceed via a radical pair intermediate. It is instructive to look at this more closely. The standard MM reaction scheme is^21^

1 $$\begin{gathered} {\text{E}}\; + \;{\text{S}}\;\;\underset{{k_{b} }}{\overset{{k_{a} }}{\rightleftharpoons}}\;{\text{ES}} \\ {\text{ES}}\;\;\mathop{\longrightarrow}\limits^{{k_{c} }}\;\;{\text{E + P,}} \\ \end{gathered}$$

where E, S, ES and P are, respectively, the enzyme, the substrate, the enzyme–substrate complex and the reaction product, and *k*_*a*_, *k*_*b*_, *k*_*c*_ are rate constants. Applying the conventional steady-state approximation to the short-lived intermediate, ES, gives the following expression for *V*, the overall rate of the reaction^21^:

2 $$V\;\; = \;\;\frac{{{\text{d}}P}}{{{\text{d}}t}}\;\; = \;\;\frac{{V_{{{\text{max}}}} S}}{{S + K_{{\text{M}}} }},$$

where

3 $$V_{{{\text{max}}}} \;\; = \;\;k_{c} E_{0} \;\;\;{\text{and}}\;\;\;K_{{\text{M}}} \;\; = \;\;\frac{{k_{b} + k_{c} }}{{k_{a} }}.$$

*P* and *S* are the concentrations of product and substrate, and *E*_0_ is the total enzyme concentration.

Suppose that switching on a weak magnetic field, or slightly changing the strength of an already-present magnetic field, produces a change $\delta k_{i}$ in one of the rate constants ($i \in a,b,c$) which in turn leads to a change $\delta V$ in the overall rate. We can then define an ‘amplification factor’, $R_{i}$, as the fractional change in the rate ($\delta V/V$) divided by the fractional change in the rate constant ($\delta k_{i} /k_{i}$) which, for small $\delta k_{i}$ is given by:

4 $$R_{i} \;\; = \;\;\left| {\frac{\delta V/V}{{\delta k_{i} /k_{i} }}} \right|\;\; \approx \;\;\left( {\frac{{k_{i} }}{V}} \right)\;\left| {\,\left( {\frac{\partial V}{{\partial k_{i} }}} \right)_{{k_{j \ne i} }} } \right|.$$

Amplification of the change in $k_{i}$ occurs when $R_{i}$ is greater than 1. Combining Eqs. (2)–(4) gives:

5 $$R_{a} \;\; = \;\;\frac{{K_{{\text{M}}} }}{{S + K_{{\text{M}}} }}.$$

$R_{a}$, therefore, clearly cannot exceed 1. The same is true of *R*_*b*_ and *R*_*c*_. There is therefore no possibility of amplification in the MM reaction scheme: a 1% change in one of the rate constants can never produce any change greater than 1% in the overall rate.

Nevertheless, amplification may be possible for more complex multi-step reaction schemes, in particular those that include chemical feedback and autocatalysis and can, as a result, exhibit chemical oscillations^22,23^. If the concentration of a chemical species affects the rate of its own production as, for example, in reactions of the form

6 $${\text{A}} + {\text{B}}\;\; \to \;\;2{\text{B}}\;\;\;{\text{and}}\;\;\;{\text{A}} + 2{\text{B}}\;\; \to \;\;3{\text{B,}}$$

then there may be a greater chance that small changes in rate constants translate into large changes in concentrations or rates. We report here a study of this possibility using a model chemical oscillator, the Brusselator.

### Theory

Our study of chemical amplification of weak magnetic field effects uses the model developed by Prigogine and Lefever in 1968^24^ in which reactants A and B are converted to products D and E via intermediate species X and Y in four interlinked reaction steps, the second of which is autocatalytic:

7 $$\begin{array}{*{20}l} {{\text{A}}\;\;\xrightarrow{{k_{1} }}\;\;{\text{X}}} \hfill \\ {{\text{Y}} + 2{\text{X}}\;\;\xrightarrow{{k_{2} }}\;\;3{\text{X}}} \hfill \\ {{\text{X}} + {\text{B}}\;\;\xrightarrow{{k_{3} }}\;\;{\text{D}} + {\text{Y}}} \hfill \\ {{\text{X}}\;\;\xrightarrow{{k_{4} }}\;\;{\text{E}}{\text{.}}} \hfill \\ \end{array}$$

The associated rate equations for X and Y are:

8 $$\begin{aligned} \frac{{{\text{d}}X}}{{{\text{d}}t}}\;\; & = \;\;k_{1} A + k_{2} X^{2} Y - k_{3} XB - k_{4} X \\ \frac{{{\text{d}}Y}}{{{\text{d}}t}}\;\; & = \;\; - k_{2} X^{2} Y + k_{3} XB, \\ \end{aligned}$$

where *A*, *B*, *X*, *Y* are the concentrations of the corresponding species and $k_{1} ,\;k_{2} ,\;k_{3} ,\;k_{4}$ are rate constants. Dubbed the ‘Brusselator’^25^, it is one of the most intensively studied chemical oscillator models^26–29^ and has been extended to reaction–diffusion systems (beyond the scope of the present study)^30–34^. Equation (8) can be recast in terms of dimensionless reduced variables^22^:

9 $$\begin{aligned} \frac{{{\text{d}}x}}{{{\text{d}}\tau }}\;\;& = \;\;a + x^{2} y - bx - x \\ \frac{{{\text{d}}y}}{{{\text{d}}\tau }}\;\; &= \;\; - x^{2} y + bx, \\ \end{aligned}$$

where

10 $$a\;\; = \;\;\frac{{k_{1} }}{{k_{4} }}\sqrt {\frac{{k_{2} }}{{k_{4} }}} A;\;\;\;\;\;b\;\; = \;\;\frac{{k_{3} }}{{k_{4} }}B;\;\;\;\;\;\frac{x}{X}\;\; = \;\;\frac{y}{Y}\;\; = \;\;\sqrt {\frac{{k_{2} }}{{k_{4} }}} ;\;\;\;\;\;\tau \;\; = \;\;k_{4} t.$$

Analysis of Eq. (9) reveals a ‘Hopf bifurcation’ at $a^{2} + 1 = b$^22,23^. At low concentrations of B, when $a^{2} + 1 > b$, the stationary-state solutions ($x_{{{\text{ss}}}} = a$ and $y_{{{\text{ss}}}} = b/a$) are stable. However, when $a^{2} + 1 < b$ the stationary state is unstable, and *x* and *y* undergo periodic oscillations. It is in this regime that we study responses to weak applied magnetic fields. Throughout, we assume that the concentrations of the reactants A and B are held constant.

Magnetic field effects can be introduced by supposing that the first step of the Brusselator model (A → X) proceeds via a radical pair (RP) which can either revert to A or proceed to X:

11 $${\text{A}}\;\;\underset{{k^{\prime}_{ - 1} }}{\overset{{k^{\prime}_{1} }}{\rightleftharpoons}}\;\;{\text{RP}}\;\;\mathop{\longrightarrow}\limits^{{k_{{\text{X}}} }}{\text{X}}{.}$$

The steps with rate constants $k^{\prime}_{1}$ and $k^{\prime}_{ - 1}$ are assumed to conserve electron spin and to take place exclusively via electronic singlet states while both singlet and triplet radical pairs can form X with the same rate constant, $k_{{\text{X}}}$. An applied magnetic field can affect the overall rate of production of X by modulating the extent and timing of coherent singlet ↔ triplet interconversion in RP and so changing the probability, $\Phi_{{\text{X}}}$, that it reacts to give X rather than returning to A^2,4,11^.

The spin dynamics of the radical pair can be described by the following master equation:

12 $$\frac{{{\text{d}}\rho \left( t \right)}}{{{\text{d}}t}}\;\; = \;\; - L\rho \left( t \right) + k^{\prime}_{1} \frac{{P^{{\text{S}}} }}{Z},$$

where the density matrix, $\rho \left( t \right)$, is defined such that its trace, ${\text{Tr}}\left[ {\rho \left( t \right)} \right]$, is equal to the concentration of radical pairs divided by the fixed concentration of A. The first term accounts for the coherent spin motion and reactivity of RP, and the second for the formation of singlet-state radical pairs from A. $P^{{\text{S}}}$ is the singlet projection operator, *Z* is the number of nuclear spin states, and the Liouvillian, *L*, is given by

13 $$L\rho \left( t \right)\;\; = \;\;i\left[ {H,\rho \left( t \right)} \right]_{ - } + \;\;\frac{{k^{\prime}_{ - 1} }}{2}\left[ {P^{{\text{S}}} ,\rho \left( t \right)} \right]_{ + } + \;\;k_{{\text{X}}} \rho \left( t \right).$$

*H* is the spin Hamiltonian which in general contains terms for the Zeeman, hyperfine, exchange and dipolar interactions of the radical pair. The second and third terms in Eq. (13) account for the RP → A and RP → X reactions, respectively. Considering RP to be a short-lived intermediate, present at low concentration (i.e. $k^{\prime}_{ - 1} + k_{{\text{X}}} > > k^{\prime}_{1}$), we can set ${\text{d}}\rho /{\text{d}}t = 0$, to obtain the steady state solution:

14 $$\rho \;\; = \;\;\frac{{k^{\prime}_{1} }}{Z}L^{ - 1} P^{{\text{S}}} .$$

Hence the rate of formation of X is:

15 $$\frac{{{\text{d}}X}}{{{\text{d}}{\kern 1pt} t}}\;\; = \;\;k_{{\text{X}}} RP\;\; = \;\;k_{{\text{X}}} {\text{Tr}}\left[ \rho \right]A\;\; = \;\;\frac{{k^{\prime}_{1} k_{{\text{X}}} }}{Z}{\text{Tr}}\left[ {L^{ - 1} P^{{\text{S}}} } \right]A,$$

which can be rewritten:

16 $$\frac{{{\text{d}}X}}{{{\text{d}}{\kern 1pt} t}}\;\; = \;\;k^{\prime}_{1} \,\Phi_{{\text{X}}} \,A\;\; = \;\;k_{1} A,$$

where:

17 $$\;\Phi_{{\text{X}}} \;\; = \;\;\frac{{k_{{\text{X}}} }}{Z}{\text{Tr}}\left[ {L^{ - 1} P^{{\text{S}}} } \right].$$

We can therefore model the effect of applied magnetic fields on the kinetics of the A → X reaction step simply by modifying the overall rate constant, $k_{1}$, and hence *a* in Eq. (9).

To explore the effects of small field-induced changes in *a* on the time-dependence of *x* and *y*, the rate equations in Eq. (9) were integrated nusing the built-in function NDSolve in *Mathematica*^35^.

## Results

### Limit cycles

**Figure 1:**
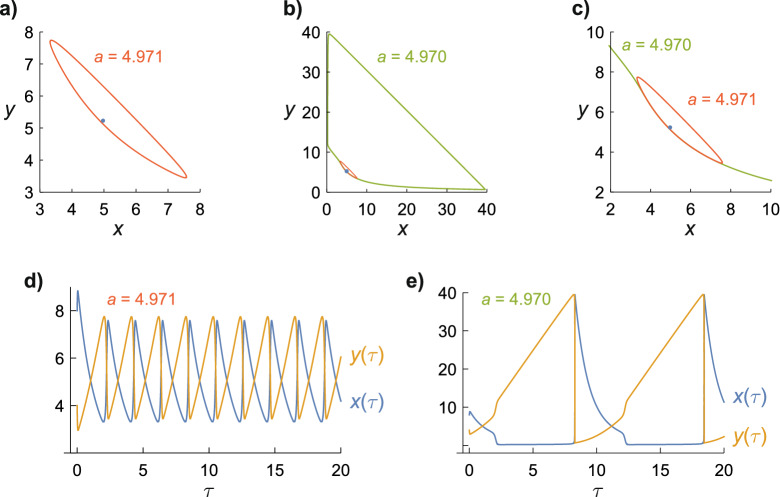
Oscillations in the concentrations of the Brusselator intermediates, $x(\tau )$ and $y(\tau )$, shown as limit cycles (**a**–**c**) and explicit time-dependence (**d,e**). The values of *a* are as indicated; *b* = 26 in all five panels. The blue dot in (**a**–**c**) is the stationary-state solution for *a* = 4.971, *b* = 26. (**c**) is an expanded view of (**b**). The initial conditions are *x*(0) = 8, *y*(0) = 4. The limit cycles do not include the induction period (*τ* < 5) required to establish stable oscillations.

We start by investigating the Hopf bifurcation at $a = 5$, $b = 26$ to see whether small changes in the value of *a*, as might arise from a weak magnetic field effect on the first step of the Brusselator model, could produce disproportionate changes in the concentrations of the intermediates. When 4.971 ≤ *a* < 5.000, $x(\tau )$ and $y(\tau )$ repeatedly traverse the same closed path, known as a limit cycle^22,23^. For example, when *a* = 4.971, $x(\tau )$ and $y(\tau )$ oscillate periodically between ~ 3.4 and ~ 7.7, encircling the stationary-state solution at $x_{{{\text{ss}}}}$ = 4.971, $y_{{{\text{ss}}}}$ = 5.230 (Fig. 1a). Unexpectedly, and remarkably, a 0.02% reduction in *a*, from 4.971 to 4.970, produces a dramatic ~ ninefold increase in the amplitude of the limit cycle (Fig. 1b), such that $x(\tau )$ and $y(\tau )$ now vary between ~ 0.4 and ~ 39.5. The two limit cycles intersect in the region of the stationary-state solution (Fig. 1c). The time-dependence of the two cycles is shown in Fig. 1d,e. The large cycle (*a* = 4.970) oscillates ~ 5 times slower than the small cycle (*a* = 4.971).

### Canard explosion

**Figure 2:**
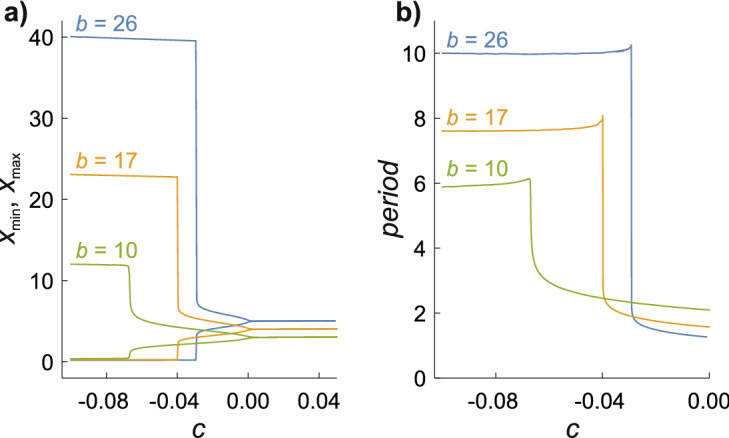
Amplitudes and periods of the Brusselator limit cycles as a function of $c = a - \sqrt {b - 1}$ for *b* = 10, 17, and 26. The Hopf bifurcation occurs at *c* = 0 and oscillatory behaviour is found for *c* < 0. (**a**) Maximum and minimum values of $x(\tau )$. The corresponding graph for $y(\tau )$ is very similar. (**b**) The oscillation period.

Further properties of this extraordinary sensitivity of the limit cycle to *a* can be seen in Fig. 2. Figure 2a shows the maximum and minimum values of *x* as a function of $c = a - \sqrt {b - 1}$ for three values of *b*. When *c* > 0 (i.e. $a^{2} + 1 > b$) there are no oscillations and *x* = *x*_ss_ = *a*. Oscillations appear on the other side of the Hopf bifurcation (*c* < 0); the limit cycle gradually expands as *a* is reduced until a step change occurs to a much larger cycle. The changes in $x_{{{\text{max}}}}$ and $x_{\min }$ at this point are sharper and more pronounced the larger the value of *b*. When *b* = 26, for example, a 2 parts per million reduction in *a* from 4.97087 to 4.97086 changes $x_{{{\text{max}}}} - x_{\min }$ from 5.2 to 39.3 and $y_{{{\text{max}}}} - y_{\min }$ from 5.3 to 38.8. Dramatic changes also occur in the oscillation period (Fig. 2b): as noted above, the larger cycle has a lower frequency.

The sudden switch from low-amplitude, fast oscillations to high-amplitude, slow oscillations is an example of a false bifurcation, known as a *canard*^36–39^. Canard explosions, as they are also known, have been found for many other oscillator models^40–42^ and for some real chemical oscillators^42^. Somewhat surprisingly, given the popularity of the Brusselator, this canard appears not to have been reported previously^39,43,44^. We stumbled across it by chance^45,46^.

### Static magnetic fields

**Figure 3:**
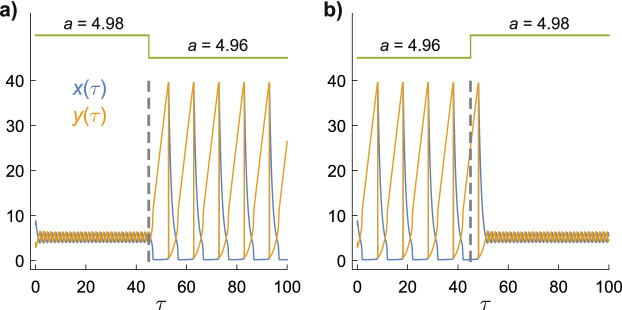
Time-dependence of the concentrations $x(\tau )$ and $y(\tau )$ when small, sudden changes are made in the value of *a*. *b* = 26 for both panels. (**a**) *a* = 4.98 → *a* = 4.96. (**b**) *a* = 4.96 → *a* = 4.98. The initial conditions are *x*(0) = 8, *y*(0) = 4. The green lines at the top indicate the changes in *a*. The vertical dashed line at *τ* = 45 marks the time at which *a* is changed.

The extreme sensitivity of $x(\tau )$ and $y(\tau )$ to *a* in the neighbourhood of the canard implies, at least in theory, that the feedback inherent in the Brusselator model could afford strong amplification of magnetic field effects on the rate constant of the A → X reaction step. Figure 3 shows solutions of Eq. (9) in which *a* is suddenly stepped from one side of the canard to the other to model the change in $k_{1}$ that might be caused by switching on or off a weak static magnetic field, or making a small increase or decrease in the intensity of an existing static magnetic field. With *b* = 26 and *a* = 4.98 (Fig. 3a), $x(\tau )$ and $y(\tau )$ move quickly from their initial values to the small limit cycle where they oscillate until *τ* = 45 at which time *a* is abruptly reduced to 4.96, provoking a switch to the large limit cycle. The reverse behaviour (Fig. 3b) is found when *a* is increased from 4.96 to 4.98 at *τ* = 45. The switching between limit cycles occurs at their intersection (*x* ≈ *y* ≈ 5, Fig. 1c), hence the delay in the transition from the slow cycle to the fast one in Fig. 3b. Figure 3 shows that large changes in the oscillation amplitudes and period can be induced by small (here ± 0.4%) changes in *a*.

### Oscillating magnetic fields

**Figure 4:**
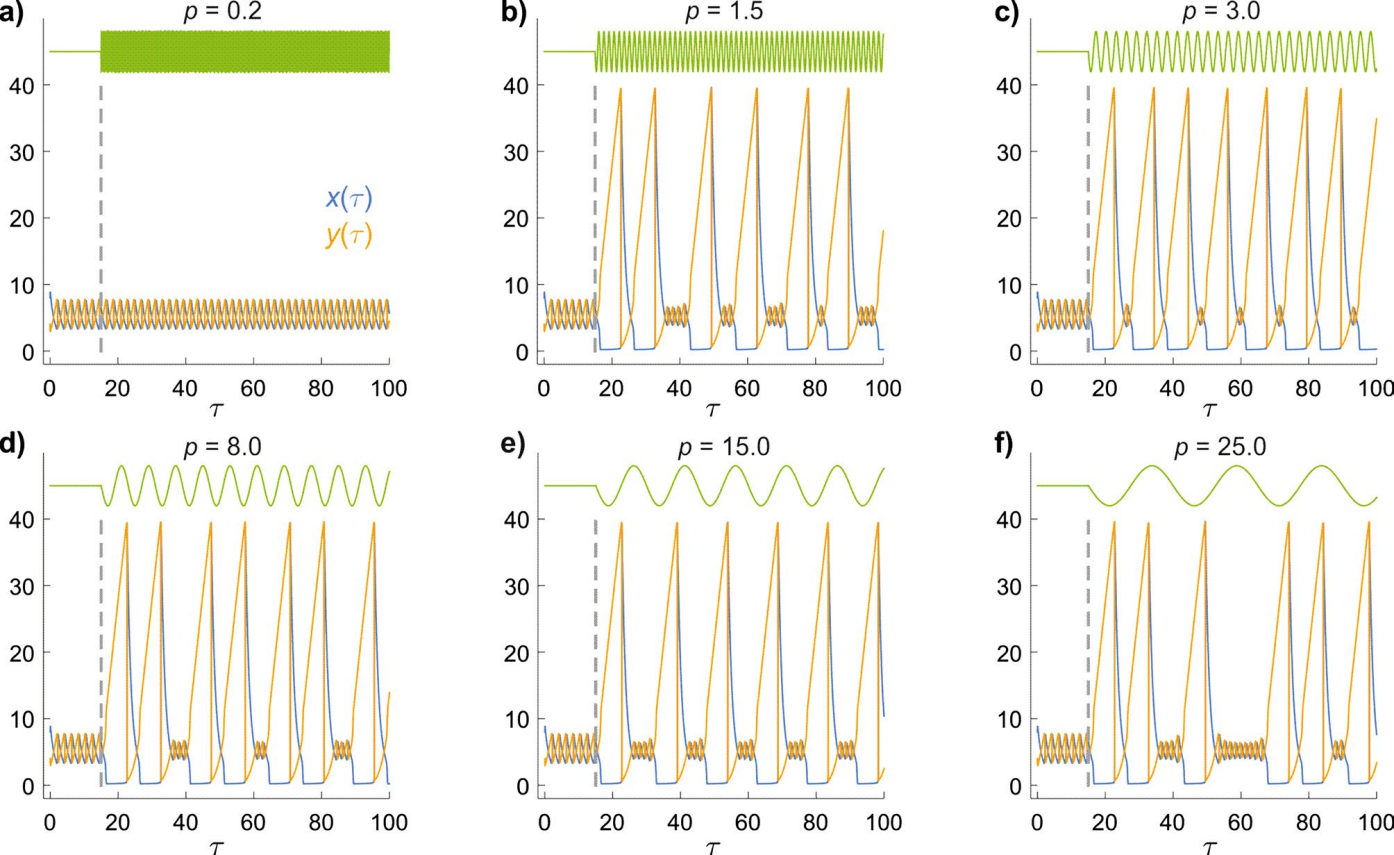
Time-dependence of the concentrations $x(\tau )$ and $y(\tau )$ when *a* is sinusoidally modulated at different frequencies. *b* = 26 for all six panels. The periods, *p*, of the oscillations are as indicated. The green lines at the top indicate the changes in *a*. The vertical dashed line at *τ* = 15 marks the time at which the modulation is switched on.

To shed light on the effects of time-dependent magnetic fields, Fig. 4 shows how $x(\tau )$ and $y(\tau )$ respond to a small sinusoidal variation in *k*_1_, and therefore in *a*. At *τ* = 15, *a* is changed from 4.971 to

18 $$a\;\; = \;\;4.971\;\; - \;\;0.01\,\sin \left( {2\pi \tau /p} \right),$$

where *p* is the period of the modulation. When *p* exceeds ~ 0.5, the initial decrease in *a* pushes $x(\tau )$ and $y(\tau )$ from the small cycle to the large cycle. Subsequently, the system switches between the two limit cycles depending on the value of *a* at the moment the concentrations reach the intersection point (Fig. 1c). For periods less than about 0.5, the system remains in the small limit cycle, presumably because *a* changes too rapidly to allow a transition to take place at the intersection point.

### Noise-modulated magnetic fields

**Figure 5:**
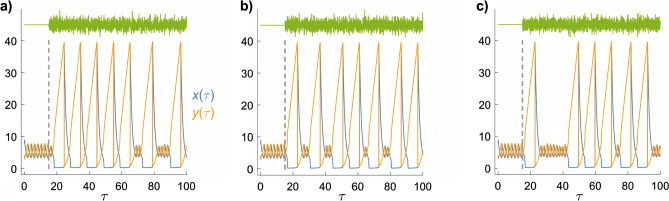
Time-dependence of the concentrations $x(\tau )$ and $y(\tau )$ when *a* is noise-modulated. *b* = 26 for all 3 panels. (**a**–**c**) Different noise realizations. The green traces at the top indicate the changes in *a*. The vertical dashed line at *τ* = 15 marks the time at which the modulation is switched on.

Next we look at the effect of noise modulation of *a* as a way of modelling a randomly varying magnetic field. In Fig. 5, at *τ* = 15, *a* is changed from 4.971 to

19 $$a\;\; = \;\;4.971\;\; + \;\;10^{ - 3} \;\sum\limits_{n = 1}^{100} {\sin \left( {2\pi \,f_{n} \tau + \phi_{n} } \right)} ,$$

where the frequencies $f_{n}$ and phases $\phi_{n}$ are random numbers with uniform probability distributions in the ranges $\{ 0.01,\;10\}$ and $\{ 0,\;2\pi \}$, respectively. With this choice of parameters, *a* has the same standard deviation (≈ 0.0071) as in Fig. 4 and Eq. (18). The 3 noise realizations shown in Fig. 5 all cause the system to switch back and forth between limit cycles, as found in Fig. 4.

### Other conditions

**Figure 6:**
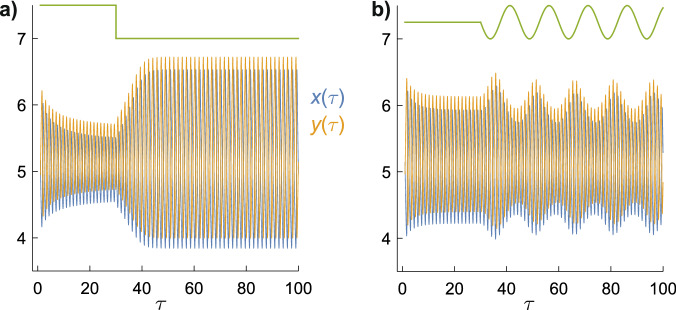
Time-dependence of the concentrations $x(\tau )$ and $y(\tau )$ when (**a**) *a* is abruptly decreased from 4.995 to 4.975, and (**b**) when *a* is sinusoidally modulated: *a* = 4.985 → *a* = 4.985 − $0.01\,\sin \left( {2\pi \tau /15} \right)$. *b* = 26 for both panels. The green lines at the top indicate the changes in *a.*

Finally, disproportionate changes in the oscillation amplitudes are not restricted to changes in rate constants that span the canard. Figure 6 shows the effect of small changes in *a* in the region between the canard and the Hopf bifurcation, visible as the − 0.03 < *c* < 0 section of the *b* = 26 trace in Fig. 2a. Although there is no abrupt change in the amplitudes as seen in Figs. 3 and 4, there is still a strong non-linear response both when *a* is abruptly decreased (Fig. 6a) and when it is sinusoidally modulated (Fig. 6b). For example, in Fig. 6a a 0.4% reduction in *a* produces a ~ 170% increase in the amplitude of the oscillations. This should be contrasted to the behaviour when oscillations are not expected, i.e. on the right hand side of the Hopf bifurcation in Fig. 2a. For example, a 0.4% change in *a*, from 5.005 to 5.0025 would produce a 0.4% change in *x* (because *x* = *x*_ss_ = *a* when $a^{2} + 1 > b$).

## Discussion and conclusions

The effects of weak magnetic fields on a chemical reaction that proceeds via a radical pair intermediate can be modelled phenomenologically by means of a field-dependent rate constant, Eqs. (16) and (17). Freed from having to consider coherent spin dynamics and spin-selective reactivity, this device has allowed us to explore in a relatively simple fashion whether autocatalysis and chemical feedback could give rise to amplification of weak magnetic field effects. Focussing on the model chemical oscillator known as the Brusselator, we have seen that dramatic changes in oscillation amplitudes can result from a minute change in a rate constant if that change spans a canard explosion (Figs. 3, 4, 5). More modest, but still substantial effects can be found under other, less strict, conditions (Fig. 6). In all cases, the relative change in the concentrations of reaction intermediates can greatly exceed the relative change in the magnetically sensitive rate constant. Defined in this way, tiny magnetic field effects can be hugely amplified in suitably autocatalytic multi-step reactions.

It is not clear whether chemical or biochemical oscillators exist that could show the kinds of amplification discussed here. The scientific literature on oscillating reactions conducted in magnetic fields is sparse to say the least. Olsen et al.^47–49^ have reported effects of strong (~ 100 mT) magnetic fields on the oscillating peroxide-oxidase reaction which they attributed to a radical pair mechanism. Eichwald and Walleczek^50^ have modelled magnetic field effects on a system of two coupled enzyme reactions in which the substrate of each reaction is the product of the other, and one reaction step had a radical pair intermediate. There have also been several studies of magnetic field effects on the celebrated Belousov-Zhabotinsky (BZ) reaction in which radicals (${\text{BrO}}_{2}^{ \cdot }$ and possibly ${\text{BrO}}^{ \cdot }$^51^) are essential intermediates and paramagnetic metal ions may be involved. Changes in wavefront velocity in unstirred BZ reactions subject to magnetic field gradients have been attributed to magnetic convection^52–56^. Related effects have been found for a travelling wave in the reaction of a Co(II) complex with hydrogen peroxide^57–60^. Blank has attributed changes in oscillation frequencies to magnetic field effects on electron transfer rates^61^ although Sontag found no effects of low frequency electromagnetic fields^62^. In none of these studies of the BZ reaction have radical pairs been implicated.

Although amplification can arise from sinusoidally varying magnetic fields (Figs. 4, 6), we have found nothing to suggest that it could exceed the amplification produced by static magnetic fields of comparable amplitude (Figs. 3, 6). At least in the case of the Brusselator, there is no evidence for resonant effects in which, hypothetically, modulating a rate constant at the frequency of the chemical oscillation would selectively drive it to higher amplitude. The simulations presented here, therefore, cannot help understand how a biological system could be more sensitive to extremely low frequency (ELF, e.g. 50/60 Hz) electromagnetic fields than to static fields of similar strength^9^. Although other model oscillators might display resonant pumping, this seems an improbable source of fortuitous ELF field effects in biology, requiring as it does a biological oscillator with a suitable radical pair intermediate, an appropriate frequency and, probably, some phase coherence between the oscillator and the ELF field^10^.
